# HOTSPOT: hierarchical host prediction for assembled plasmid contigs with transformer

**DOI:** 10.1093/bioinformatics/btad283

**Published:** 2023-04-22

**Authors:** Yongxin Ji, Jiayu Shang, Xubo Tang, Yanni Sun

**Affiliations:** Department of Electrical Engineering, City University of Hong Kong, Hong Kong (SAR), China; Department of Electrical Engineering, City University of Hong Kong, Hong Kong (SAR), China; Department of Electrical Engineering, City University of Hong Kong, Hong Kong (SAR), China; Department of Electrical Engineering, City University of Hong Kong, Hong Kong (SAR), China

## Abstract

**Motivation:**

As prevalent extrachromosomal replicons in many bacteria, plasmids play an essential role in their hosts’ evolution and adaptation. The host range of a plasmid refers to the taxonomic range of bacteria in which it can replicate and thrive. Understanding host ranges of plasmids sheds light on studying the roles of plasmids in bacterial evolution and adaptation. Metagenomic sequencing has become a major means to obtain new plasmids and derive their hosts. However, host prediction for assembled plasmid contigs still needs to tackle several challenges: different sequence compositions and copy numbers between plasmids and the hosts, high diversity in plasmids, and limited plasmid annotations. Existing tools have not yet achieved an ideal tradeoff between sensitivity and precision on metagenomic assembled contigs.

**Results:**

In this work, we construct a hierarchical classification tool named HOTSPOT, whose backbone is a phylogenetic tree of the bacterial hosts from phylum to species. By incorporating the state-of-the-art language model, Transformer, in each node’s taxon classifier, the top-down tree search achieves an accurate host taxonomy prediction for the input plasmid contigs. We rigorously tested HOTSPOT on multiple datasets, including RefSeq complete plasmids, artificial contigs, simulated metagenomic data, mock metagenomic data, the Hi-C dataset, and the CAMI2 marine dataset. All experiments show that HOTSPOT outperforms other popular methods.

**Availability and implementation:**

The source code of HOTSPOT is available via: https://github.com/Orin-beep/HOTSPOT

## 1 Introduction

Plasmids refer to extrachromosomal replicons found in microorganisms across three superkingdoms, *Bacteria* (mostly), *Archaea*, and *Eukaryota* (Lederberg 1952). They are usually circular DNA molecules with varying sizes from 744 to 2.58 Mb (megaplasmids) (Shintani et al. 2015). Plasmids naturally occur in a large proportion of bacteria and play a critical role in the evolution of bacteria. Specifically, plasmids can mediate horizontal gene transfer between bacterial cells through conjugation (Shintani and Suzuki 2019). As a result, genetic elements carrying traits such as antibiotic resistance, virulence, and enzymes involved in secondary metabolism (Rankin et al. 2011), can spread in bacterial communities. These traits are generally non-essential but can enhance the host’s fitness for new or changed environments. Predicting the host association of plasmids will contribute to detecting the spread of plasmids in environmental bacterial communities and understanding how plasmids facilitate the evolution and adaptation of bacteria, especially human pathogens. Moreover, it is also significant to the artificial construction of plasmids as vector tools in genetic engineering (Jacob and Grinter 1975).

The host range of a plasmid is defined as the various microorganisms by which it can be hosted (replicated, maintained, and transferred). Depending on the taxonomic breadth of the potential host bacteria, plasmids can be divided into narrow-host-range (NHR) and broad-host-range (BHR) (Jain and Srivastava 2013). The hosts of BHR plasmids are phylogenetically distant organisms, such as bacteria belonging to different families, classes, or even phyla. In contrast, NHR plasmids may only be hosted by bacteria from the same species. It is reported that most of the plasmids identified until now are NHR (Smorawinska et al. 2012), and the studies on BHR plasmids are limited. Lots of evidence shows that the plasmid host range is associated with replication and conjugation, which can be characterized by three factors: the types of replicon, mobility (MOB), and mating pair formation (MPF). To predict the three types, we can use BLAST to compare the query plasmid against reference databases containing conserved replicon sequences, relaxases of nine MOB families, and MPF proteins of four MPF systems (Carattoli et al. 2014; Shintani et al. 2015; Garcillán-Barcia et al. 2020). However, the current replicon database can only work for plasmids within a restricted taxonomic range, and the MOB and MPF databases do not contain proteins for non-mobilizable plasmids. As a result, these typing methods are not sufficient for host prediction. Besides, Acman et al. (2020) show that 72.1% of the plasmid proteins have no functional annotation, which increases the difficulty of using these plasmid types for host range prediction.

The plasmid host can be tested with selected recipient strains through traditional culture-dependent methods under appropriate conditions (Pukall et al. 1996). Some culture-independent techniques were also developed to analyze the *in situ* plasmid host range (Klümper et al. 2015). In particular, next-generation sequencing and its application in metagenomics have revealed a large number of novel plasmids. For example, the number of complete plasmid sequences in the NCBI RefSeq database was expanded from 10 634 in November 2017 to 38 277 in June 2022. The fast growth of the data makes computational host prediction a promising alternative to experimental methods. Recently, many tools have been presented to classify plasmid- or chromosome-borne contigs for metagenomic assemblies (Robertson and Nash 2018, Schwengers et al. 2020), facilitating the host prediction for the identified plasmid contigs.

There are three challenges for plasmid host prediction in metagenomic data. First, different sequence compositions and copy numbers between the chromosomes and plasmids hinder contig binning tools from grouping plasmid contigs with their host chromosomes (Maguire et al. 2020). Based on our BLAST experiments, 23 465 out of 30 494 (∼77%) complete plasmids have alignment coverage below 10% against their host chromosomes (with percent identity >80%). Although SMRT sequencing succeeds in finding the plasmid–host links via the methylated base occurrence locations (Beaulaurier et al. 2018), it cannot be generalized to most available datasets. Thus, generic contig binning tools cannot be applied for host prediction. Second, plasmids with the same host range can also exhibit high diversity, posing a challenge for alignment-based host prediction. Our experiments show that the average Dashing (Baker and Langmead 2019) similarity between 9430 plasmids of the species *Escherichia coli* (the most common plasmid host) is only 0.021. Third, the contigs assembled from metagenomic data are typically fragmented, which only carry limited information for host prediction.

### 1.1 Related work

Existing computational host prediction tools can be roughly classified into two types: alignment-based and machine learning-based models. The alignment-based methods follow the rationale that plasmids with high genetic similarity tend to have the same host range. Thus, their workflows are mainly based on the comparison between query plasmids and plasmids with known hosts. For example, MOB-suite (Robertson and Nash 2018) includes a module called MOB-typer (Robertson et al. 2020) for host prediction via Mash-based clustering (Ondov et al. 2016). The authors in (Redondo-Salvo et al. 2020) proposed an algorithm to organize highly similar plasmids into the same cluster called plasmid taxonomic unit (PTU). Accordingly, COPLA (Redondo-Salvo et al. 2021) was developed to assign each input plasmid to an existing PTU. The experiments showed that most plasmids in the same PTU have identical host ranges. Acman et al. (2020) implemented Ordered Statistics Local Optimization Method (OSLOM), a community detection algorithm based on Jaccard similarity to cluster plasmids into complete subgraphs. The results revealed that the generated subgraphs correlate highly with the bacterial hosts. However, the alignment-based methods suffer from two main limitations. First, novel plasmids may not have significant alignments with the curated plasmids or proteins, leading to low sensitivity for host prediction. Second, when predicting the hosts using network-based clustering methods such as COPLA (PTUs) and OSLOM, the query plasmid needs to be compared with all plasmids in the database by rebuilding the network, which is time-consuming. For example, COPLA needs around 5 min to assign PTU to one input plasmid.

Two machine learning-based tools have been proposed to overcome the limitations of alignment-based methods. These tools usually formulate the host prediction as multiclass classification problems at specific taxonomic ranks. They mainly adopt *k*-mers, GC-content, and codon usage as features. For example, PlasmidHostFinder (Aytan-Aktug et al. 2022) utilizes plasmid genomic signatures, such as 8-mer counts and codon frequencies, to train four random forest models corresponding to the order, family, genus, and species level. PlasFlow (Krawczyk et al. 2018) trained neural networks using normalized *k*-mer frequency vector to predict phylum-level plasmid hosts. A common challenge for machine learning-based host prediction is the expanded label set and decreased training data for lower ranks, such as genus. Current tools did not address this challenge well, which is part of the reason behind their decreased performance on real metagenomic data.

### 1.2 Overview

In this work, we present a tool named HOTSPOT, which formulates plasmid host prediction as a hierarchical taxonomic classification problem from phylum to species. The backbone of HOTSPOT is the bacterial hosts’ phylogenetic tree, with each node being a classifier for its child nodes. The hierarchical structure has a unique advantage of returning a higher-rank host taxon for a novel plasmid when its host taxa at lower ranks do not exist yet in the training data. In addition, each node-based classifier only distinguishes its child nodes rather than all taxa at a specified rank as designed in current tools. Because various proteins play vital roles in determining the host range, we represent plasmids as a language defined on a protein token set and exploit the state-of-the-art language model, Transformer (Vaswani et al., 2017), to learn the importance of proteins and their associations. In each classifier, we take all computationally predicted proteins (CPs), annotated MOB/MPF proteins, and identified incompatibility (Inc) group (by replicon typing) of the query plasmid contig as input features for taxon classification. We compared our tool with the state-of-the-art approaches designed for plasmid host prediction: MOB-typer (Robertson et al. 2020), PlasmidHostFinder (Aytan-Aktug et al. 2022), and PlasFlow (Krawczyk et al. 2018). We rigorously tested our tool on multiple datasets, including RefSeq complete plasmids, artificial contigs, mock metagenomic data, the Hi-C dataset, and the CAMI2 marine dataset. The experimental results demonstrated that HOTSPOT outperforms other popular methods on all the datasets.

## 2 Methods

Given a plasmid contig as input, HOTSPOT conducts hierarchical search from the root node to lower ranks to predict its host’s taxon. In general, the difficulty of host prediction increases at lower ranks, such as genus and species, because fewer samples are associated with them. Thus, our tree search allows early stop when the prediction uncertainty based on Monte Carlo Dropout (MC-dropout) is above a given cutoff, enabling us to output more accurate predictions with a small sacrifice of the resolution.

In the following sections, we first introduce the tree-based backbone. Then, we will show the tokenization method and how we encode the plasmid contigs into sentences. Third, we will describe the Transformer-based model optimized for the host taxon classification on each node. Finally, we will detail the early stop mechanism using MC-dropout.

### 2.1 The hierarchical classification framework

**Figure 1.**
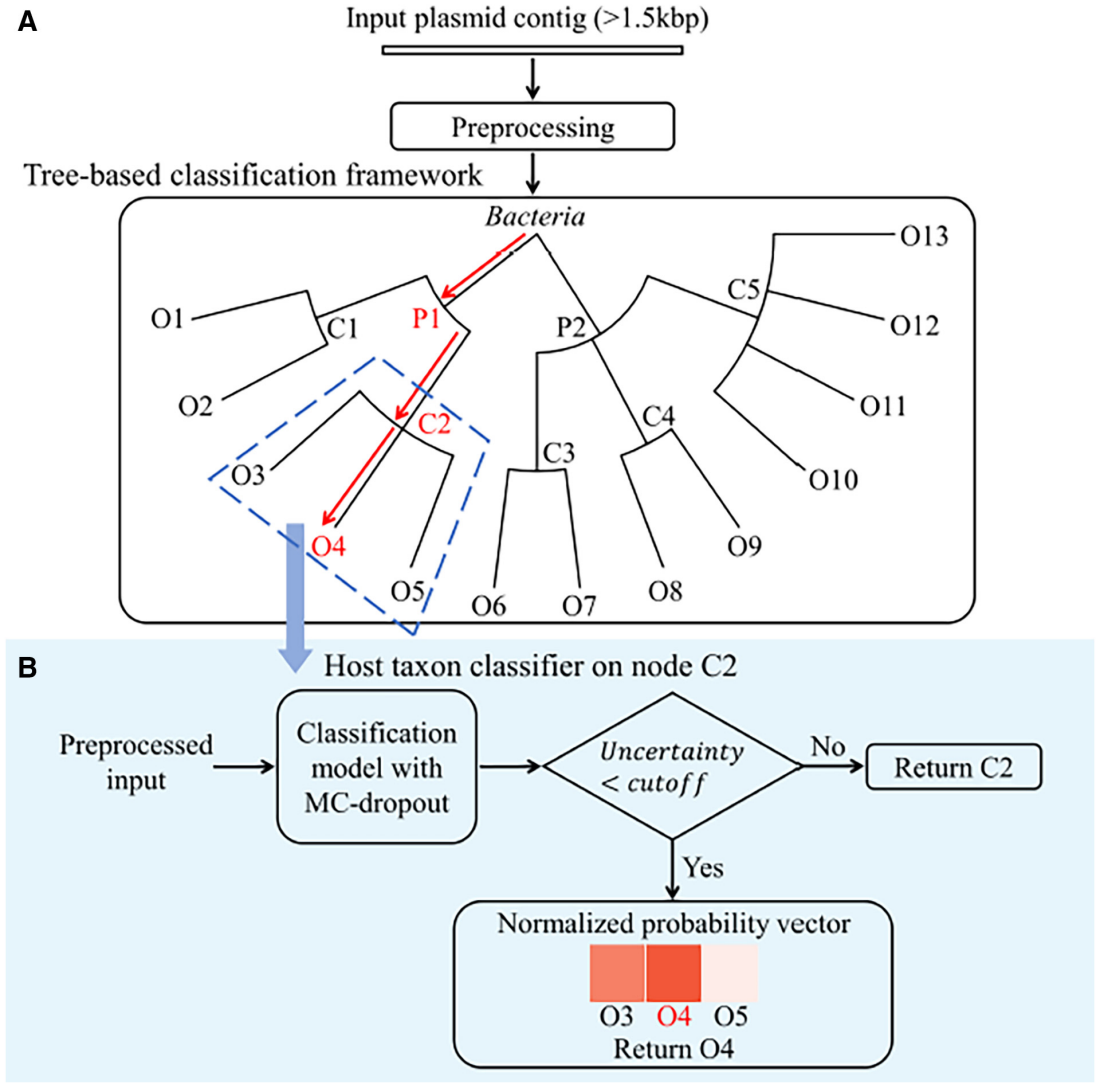
The pipeline of HOTSPOT. (A) The tree-based backbone of HOTSPOT. The inputs are identified plasmid contigs with a minimum length of 1.5 kb. Preprocessing is detailed in Fig. 2. Due to space limitation, we only plot part of the tree containing phylum, class, and order levels. The nodes annotated with $P_{i}$, $C_{i}$, and $O_{i}$ represent phylum, class, and order labels, respectively. (B) The classifier for class *C*2. Based on the estimated model uncertainty, it either returns an identified taxon in the next rank or stops early by returning *C*2.

Figure 1A sketches the hierarchical classification framework, which is a phylogenetic tree for known plasmid hosts. Its depth is 7, consisting of the root node *Bacteria* and the six ranks from phylum to species. This tree is smaller than the complete bacterial phylogenetic tree because: (i) not all bacteria contain plasmids and (ii) the host taxa covered by available sequenced plasmids are limited. Each node is a classifier with the label set *Y* as the taxa of its child nodes. For example, the classifier for node *C*2 (a class) in Fig. 1B will classify an input into one of the three taxa, *O*3, *O*4, and *O*5 (orders). If the classifier returns high prediction uncertainty, the early stop mechanism allows HOTSPOT to return *C*2 rather than continuing to go deeper in the tree. The uncertainty is estimated using MC-dropout (Fig. 1B).

### 2.2 Transformer-based classifier

**Figure 2.**
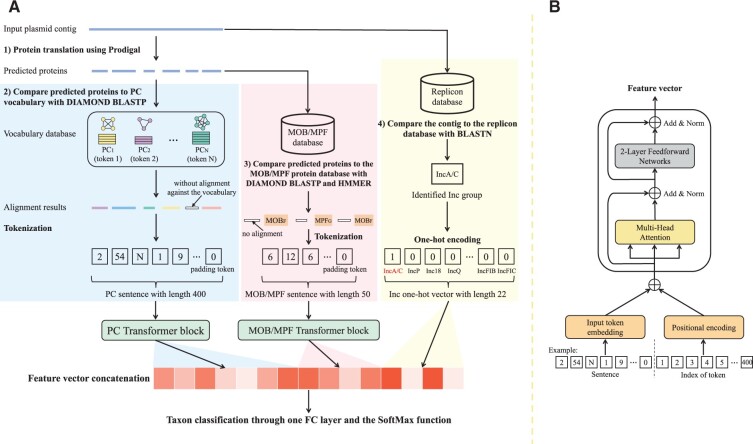
(A) The workflow of the preprocessing and the Transformer-based classifier. The blocks with blue, red, and yellow backgrounds represent three preprocessing sections: PC sentence construction, MOB/MPF sentence construction, and identified Inc group encoding, respectively. The feature vectors extracted from the two Transformer blocks and the Inc one-hot vector will be concatenated for the final taxon classification. (B) The architecture of the Transformer block, which consists of a self-attention layer and a two-layer neural network. The input of the Transformer block is the sum of the embedded sentence and the embedded positional index vector.

Figure 2 illustrates the structure of each node-based classifier, which uses Transformer to learn the ‘language’ of plasmids. We employed three types of features. First, because proteins have great importance to host ranges (Shintani and Suzuki 2019), we construct protein clusters (PCs) and use them as tokens in one Transformer. Because many of these proteins have no functional annotation (Acman et al., 2020), we add the second set of features based on well-curated Inc and MOB/MPF classification information. The MOB/MPF databases contain relaxases of nine MOB families and MPF proteins of four MPF systems (Shintani et al. 2015; Garcillán-Barcia et al. 2020). The Inc database contains conserved replicon sequences to group plasmids with matching replication or partitioning systems into the same Inc group (Carattoli et al. 2014). More descriptions of these databases can be found in Supplementary Section S1. To leverage these well-annotated features, we first combine the annotated MOB/MPF proteins on the query plasmid into a new sentence as the input of another Transformer (with different hyper-parameters from the first one). Second, the identified Inc group will be encoded into a one-hot vector and participate in the final taxon classification.

#### 2.2.1 Constructing the PC sentence

##### Building the protein-cluster vocabulary

Homologous proteins are the token set (vocabulary) of the PC Transformer. To generate the PCs, we first use Prodigal (Hyatt et al. 2010) for gene identification and protein translation of all the complete plasmids we collected (the performance assessment of Prodigal is conducted in Supplementary Section S2). Second, all-against-all alignment with the maximum *E*-value 1*e*−3 (E≤0.001) is employed on all the translated CPs using DIAMOND BLASTP (Buchfink et al. 2015). The alignment results can establish a protein similarity graph, where each node represents a protein, and each edge represents the similarity’s significance ($-{\;\text{log}\;}_{10}E$). In Supplementary Section S3, we showed that using the cutoff 1*e*−3 can lead to high-quality alignments. Then, we implement the Markov Clustering Algorithm (MCL) (Enright et al. 2002) to group highly similar proteins into the same cluster. The generated PCs with at least two proteins act as tokens and jointly form a vocabulary to facilitate the PC sentence construction. As a result, we totally generated 108 274 PCs using the 30 284 curated plasmids.

##### Encoding the input plasmid contigs into PC sentences

Each contig is converted into a PC-based sentence. Its protein products predicted by Prodigal are aligned to the pre-created PC vocabulary using DIAMOND BLASTP. Each protein will be assigned to a token, which is the PC with the minimum *E*-value (E≤0.001). Additionally, each protein’s absolute position is recorded for the positional encoding. During the experiments, we observed that the assigned PC tokens rarely exceed 400 because we removed all megaplasmids (>350 kb) in advance. Therefore, the PC sentence length is fixed at 400. We will keep the first 400 tokens for sentences longer than 400 and use ${\text{token}}_{0}$ for padding at the end of sentences shorter than 400.

#### 2.2.2 Constructing the MOB/MPF sentence and Inc one-hot vector

As shown in Fig. 2A, the translated CPs will be aligned to the MOB/MPF databases using DIAMOND BLASTP and HMMER (Mistry et al. 2013) for annotation. The input contig will be aligned to the Inc database using BLASTN. The protein assigned with one MOB/MPF type will be converted to a token. As a result, there are 13 MOB/MPF tokens in total (nine MOB types plus four MPF types). Figure 2A shows that three of six translated CPs have the identified MOB/MPF types: MOB${}_{F}$, MPF${}_{G}$, and MOB${}_{F}$. Similar to the PC sentence building, the encoded MOB/MPF sentence is 6, 12, 6 followed by 47 ${\text{token}}_{0}$ (the maximum MOB/MPF sentence length is set to 50). In addition, the identified Inc type of the query plasmid will be encoded into a one-hot vector.

#### 2.2.3 Architecture of the Transformer model

The Transformer block applied in this work is the Transformer encoder, which can convert the input sentence into a latent feature vector with a fixed length. The feature vectors output by the two Transformer blocks and the Inc one-hot vector will be concatenated for the host’s taxon classification. As shown in Fig. 2B, the Transformer block can be divided into three parts: the embedding layers, the self-attention layer, and the feed-forward neural networks, which are described thoroughly in Supplementary Section S4.

##### Multi-head attention

To further improve the performance of the self-attention layer, we apply multi-head attention, which uses *h* groups (heads) of the three fully connected layers (FC layers) to learn multiple sets of query/key/value for prediction. The outputs of different heads will be concatenated and flow through one FC layer for downstream computation. Because the initialized parameters of multiple heads are random and these heads can be implemented in parallel, the multi-head attention will enhance the training stability. The multi-head attention can be defined in Equation (1). where *X* is the embedded vector generated by the embedding layers. ${\text{head}}_{i}$ is the *i*th head and we select h=8 by default in this work. The dimensions of $W_{i}^{Q}$, $W_{i}^{K}$, and $W_{i}^{V}$ are all $\text{embed}\times d_{\text{head}}$, leading to the head’s output dimension being equal to $\text{len}\times d_{\text{head}}$ ($d_{\text{head}}=\text{embed}/h$ by default). The smaller output dimension will reduce the computational complexity. In addition, the parameter matrix $W^{O}\in R^{hd_{\text{head}}\times \text{embed}}$.


(1) $$\{\begin{array}{c} {\text{head}}_{i}=\text{Attention}(XW_{i}^{Q},XW_{i}^{K},XW_{i}^{V})\\ \text{MultiHead}(Q,K,V)=\text{Concat}({\text{head}}_{1},\ldots ,{\text{head}}_{h})W^{O} \end{array},$$

### 2.3 The early stop mechanism using MC-dropout

HOTSPOT contains an early stop mechanism using MC-dropout, which can evaluate the model’s uncertainty during prediction. The early stop allows us to output an ancestor taxon for an input if (i) its low-rank taxon is not part of the current classification system or (ii) its prediction confidence is low. Following the mathematical derivation in (Gal and Ghahramani 2016), we can estimate the uncertainty of dropout deep neural networks (NNs) as shown in Equation (2). where $q(y^{*}|x^{*})$ is the approximate predictive distribution (the $y^{*}$ output by NNs) of a given input $x^{*}$. The two equations calculate the first moment and second moment of the function $E_{q(y^{*}|x^{*})}(y^{*})$, respectively. The first moment is observed to be equal to the average results generated by *T* stochastic forward passes (dropout-enabled predictions). $W_{i}^{t}$ represents the weight vector in the *i*th NN layer during the *t*th forward pass. The weight matrix varies in different forward pass processes because the dropout layers randomly set several weights to 0. *L* is the layer number of the dropout NNs. In each classifier of HOTSPOT, there are seven dropout layers (L=7), three for each Transformer block (the summed embedded vector and the two-layer feed-forward networks) and one for the concatenated feature vector. τ is a hyper-parameter to describe the model precision. Then, it is easy to calculate the model’s predictive variance ${\text{Var}}_{q(y^{*}|x^{*})}(y^{*})$ (the second central moment) based on the first and second moments (Equation 3).


(2) $$\{\begin{array}{c} E_{q(y^{*}|x^{*})}(y^{*})\approx \frac{1}{T}\sum_{t=1}^{T}{\hat{y}}^{*}(x^{*},W_{1}^{t},\ldots ,W_{L}^{t})\\ E_{q(y^{*}|x^{*})}({\left(y^{*}\right)}^{T}y^{*})\approx \tau^{-1}I_{D}+\\ \;\;\;\;\;\;\;\frac{1}{T}\sum_{t=1}^{T}{\hat{y}}^{*}{\left(x^{*},W_{1}^{t},\ldots ,W_{L}^{t}\right)}^{T}{\hat{y}}^{*}(x^{*},W_{1}^{t},\ldots ,W_{L}^{t}) \end{array},$$

(3) $$Var_{q(y^{*}|x^{*})}(y^{*})\approx E_{q(y^{*}|x^{*})}({\left(y^{*}\right)}^{T}y^{*})-E_{q(y^{*}|x^{*})}{\left(y^{*}\right)}^{T}E_{q(y^{*}|x^{*})}(y^{*})$$

**Figure 3.**
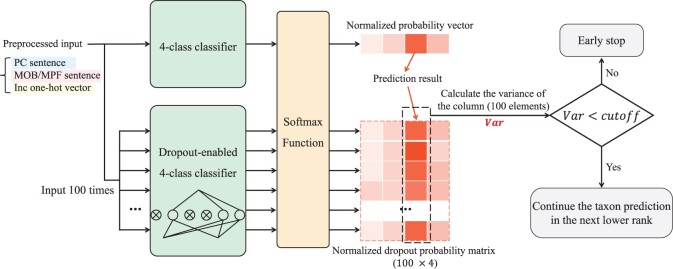
The workflow of the early stop mechanism based on MC-dropout. We use a four-class taxon classifier here for illustration.

Therefore, the model’s predictive variance depends on the variance of the *T* results output by stochastic forward passes. As shown in Fig. 3, in addition to the prediction, the input will flow through the dropout-enabled model for *T* times (T=100 by default). The sample variance of the predicted class acts as the uncertainty. If the uncertainty exceeds a given cutoff, the tree search ends, and the prediction result of the current classifier will be discarded. Users can choose whether to enable the early stop mechanism or not. Based on different uncertainty cutoffs, we create three modes for HOTSPOT: ‘sensitive’, ‘specific’, and ‘accurate’. The sensitive mode does not allow early stop. The accurate mode has a more stringent uncertainty cutoff than the specific mode, leading to more accurate prediction but returning taxa in higher levels for some inputs.

### 2.4 Data collection and model training

We collected sequenced plasmids from three sources: 36 070 complete bacterial plasmids from the RefSeq database released before June 2022, and two well-curated bacterial plasmid databases, PLSDB (Galata et al. 2019) and COMPASS (Douarre et al. 2020). The redundant genomes shared by different databases were filtered using Dashing (Baker and Langmead 2019) and complete-linkage clustering (see Supplementary Section S5). We do not consider megaplasmids in this work. Thus, the plasmids whose nucleotide lengths are greater than 350 kb were removed. The taxonomic annotation and lineage retrieval of the plasmid hosts were implemented using the Entrez Direct tool (Kans 2022) and the ETE3 Python package (Huerta-Cepas et al. 2016). Only the plasmids with complete annotated host lineages from phylum to species will be kept, which results in 30 284 plasmids for the model training. Based on these annotated lineages, we construct the phylogenetic tree of plasmid hosts. We removed the taxon nodes associated with <10 plasmids because these nodes do not have sufficient samples for training. As a result, there remain 10, 19, 46, 94, 180, and 272 taxon nodes of the six ranks from phylum to species, respectively.

For each taxon node, we first sort its plasmids by their release dates in NCBI and then separate them into training, validation, and test subsets in proportion to 70%, 15%, and 15%. We also augment the training data by randomly cutting fragments ranging from 1.5 to 10 kb from the existing training set. In this manner, the augmented data can serve as a regularizer and help prevent the overfitting by complete plasmids. We utilize cross-entropy loss function and Adam optimizer with a learning rate of 0.005 at each iteration step. The classifiers at different taxon nodes are trained individually with 50 epochs. The model training is implemented on HPC with the RTX 3090 GPU unit. Finally, the 115 pre-trained classifiers will be placed together for plasmid host prediction. The number of classifiers is less than the number of taxon nodes from phylum to genus because some nodes have only one child node. When using the HOTSPOT tool, only one pre-trained classifier is loaded into the GPU at a time, enabling successful running even on a computer with a small GPU memory.

## 3 Results

### 3.1 Experiment design

We compared HOTSPOT with three state-of-the-art tools designed for plasmid host prediction: MOB-typer (Robertson et al. 2020), PlasmidHostFinder (Aytan-Aktug et al. 2022), and PlasFlow (Krawczyk et al. 2018), all of which are briefly described in Section 1.1. PlasFlow can only output phylum-level results. MOB-typer can give predictions at the genus level or above. PlasmidHostFinder and HOTSPOT can work on all six taxonomic ranks. We add a sensitive mode for PlasmidHostFinder because its default classification probability threshold of 0.5 leads to a low recall value on metagenomic assembled contigs. When applying the sensitive mode of PlasmidHostFinder, the taxon with maximum predicted probability will be output. To have a fair comparison, we applied our training set to build the custom database for MOB-typer. We could not retrain models for PlasmidHostFinder and PlasFlow because they did not provide such functions/scripts.

As the input to HOTSPOT, the plasmid-borne contigs have to be identified from metagenomic contigs using tools such as MOB-recon (Robertson and Nash 2018) and Platon (Schwengers et al. 2020). With our testing, alignment-based tools usually achieve higher precision than *de novo* classifiers with some sacrifice of recall. Nevertheless, it is still possible for alignment-based tools to generate false positives (chromosome-borne contigs). Thus, we analyze both the performance of plasmid contig identification tools and the behavior of HOTSPOT when the inputs are chromosome-borne contigs in Supplementary Section S6. As plasmid identification requires a separate set of efforts, the following experiments focus on the performance of host prediction for input plasmids.

#### 3.1.1 Metrics

We employ prediction rate, accuracy, and F1-score as the performance metrics in each rank for a fair comparison. Their formulas are listed below (Equations 4–6):


(4) $$\;\text{prediction}\;\text{rate}=\frac{\text{number}\;\text{of}\;\text{predicted}\;\text{contigs}}{\text{number}\;\text{of}\;\text{total}\;\text{contigs}}$$

(5) $$\;\text{Accuracy}=\frac{\text{number}\;\text{of}\;\text{correctly}\;\text{predicted}\;\text{contigs}}{\text{number}\;\text{of}\;\text{predicted}\;\text{contigs}}$$

(6) $$\;F1-\text{score}=\frac{2\;*\;\text{precision}\;*\;\text{recall}}{\text{precision}+\text{recall}}=\frac{2\text{TP}}{2\text{TP}+\text{FP}+\text{FN}}.$$

Because the plasmid host prediction is a multiclass classification, we first calculate each class’s TP, FN, and FP, representing the number of correctly predicted plasmids, missed plasmids, and falsely predicted plasmids, respectively. Then, we will sum the respective TP, FN, and FP values across all classes in a rank and plug them into Equation (6) to get the F1-score. Notably, if a plasmid does not have a prediction at a rank, it will be considered an FN when we calculate the F1-score for this rank. For example, if the plasmid’s prediction stops at the family level, it will be taken as FN at the genus and species level.

#### 3.1.2 Datasets

We rigorously tested HOTSPOT on multiple datasets, including RefSeq complete genomes, artificial contigs, simulated metagenome-assembled genome (MAG) dataset, four mock metagenomic datasets, one Hi-C dataset, and one CAMI2 marine dataset. Detailed information, such as the number of high-quality plasmid contigs and the number of the hosts’ annotated taxa in the six ranks, are listed in Table 1. In some cases, such as the simulated MAG dataset, the number of species is smaller than genera because some hosts are rare and do not exist in our species-level label set. The descriptions of the datasets are listed below:

**Table 1.** The detailed information of the datasets.

Datasets	Number of plasmid contigs	Number of phyla	Number of classes	Number of orders	Number of families	Number of genera	Number of species
Complete plasmid test set	4536	10	19	46	94	180	272
Short plasmid contig test set	80 133	10	19	46	94	180	272
Simulated metagenomic dataset	106	3	4	7	8	9	8
SRR072232 (mock)	25	3	4	4	4	4	4
SRR072233 (mock)	15	3	4	4	5	5	5
SRR172902 (mock)	11	3	4	4	4	4	4
SRR172903 (mock)	21	2	2	2	2	2	2
Hi-C dataset	501	5	9	17	19	20	22
CAMI2 marine dataset	1127	6	10	22	25	29	14

- *Complete plasmid test set*: We sort the curated 30 284 plasmids by their release date and pick ∼15% of the most recently released plasmids as the test set, which contains roughly 4536 complete plasmids.
- *Short plasmid contig test set*: The shortest contig length accepted by HOTSPOT is 1.5 kb because contigs smaller than 1.5 kb may not have predicted protein-coding genes. We randomly cut the genomes in the complete plasmid test set into segments with four different lengths: 1.5, 3, 5, and 10 kb, leading to 22 078, 21 164, 19 508, 17 383 contigs for each length. The contigs’ labels are the same as their originating plasmids.
- *Simulated metagenomic dataset*: We adopt a MAG dataset, a hard case used in the review article (Maguire et al. 2020), which shows that current binning tools fail to bin plasmid contigs with their hosts. The authors simulated reads from these reference genomes, and we conducted assembly using MEGAHIT (Li et al. 2015). The reference genomes used to simulate the MAG dataset contain both plasmids and their host chromosomes, which are detailed in Supplementary Section S7.1. We screen plasmid contigs by aligning them to the provided reference plasmids (Maguire et al. 2020) and keeping ones with at least 90% identity and 90% coverage. Contigs that can be mapped to plasmids in the training set will be removed before the experiments. Due to the page limit, the results of this experiment are described in Supplementary Section S10.
- *Mock metagenomic datasets*: Four Human Microbiome Project (HMP) mock community shotgun sequencing datasets are downloaded from NCBI (accession number SRR072232, SRR072233, SRR172902, and SRR172903). The data are sequenced by either Illumina or 454 sequencing technology. Among the four datasets, two have staggered abundance distribution, and the other two have even relative abundance distribution. The authors in (Tu et al. 2014) have identified the microorganisms in these datasets, including 21 bacterial strains with 50 plasmids (for the list, see Supplementary Section S7.2). We use MEGAHIT to assemble the four datasets because they are single-end reads. Contigs that can be aligned against reference plasmids with at least 90% identity and 90% coverage are chosen as plasmid contigs and input to all the tools. Similar to the MAG dataset, the contigs that can be mapped to the plasmids in the training set will be removed first.
- *Hi-C dataset*: Hi-C sequencing can estimate the contact frequency of two DNA fragments in 3D space. Using the metagenomic Hi-C data from a wastewater community, Stalder et al. (2019) succeeded in linking plasmid contigs to their host chromosomes, resulting in 1307 groups of binned contigs. The labels of all contigs in the same bin are identical and annotated by Mash (Ondov et al. 2016) to the species level. The plasmid contigs in each bin are determined as the intersection of the identified plasmids by MOB-recon (Robertson and Nash 2018) and Platon (Schwengers et al. 2020).
- *CAMI2 marine dataset*: The CAMI2 marine datasets are complex and challenging datasets to assess various bioinformatics methods (Meyer et al. 2022). The CAMI2 datasets are computationally simulated from about 2300 known and new genomes, including many new plasmids. We evaluate our tool on the CAMI2 dataset S0. The results of this experiment are described in Supplementary Section S12.

### 3.2 Experiments on the RefSeq datasets

**Figure 4.**
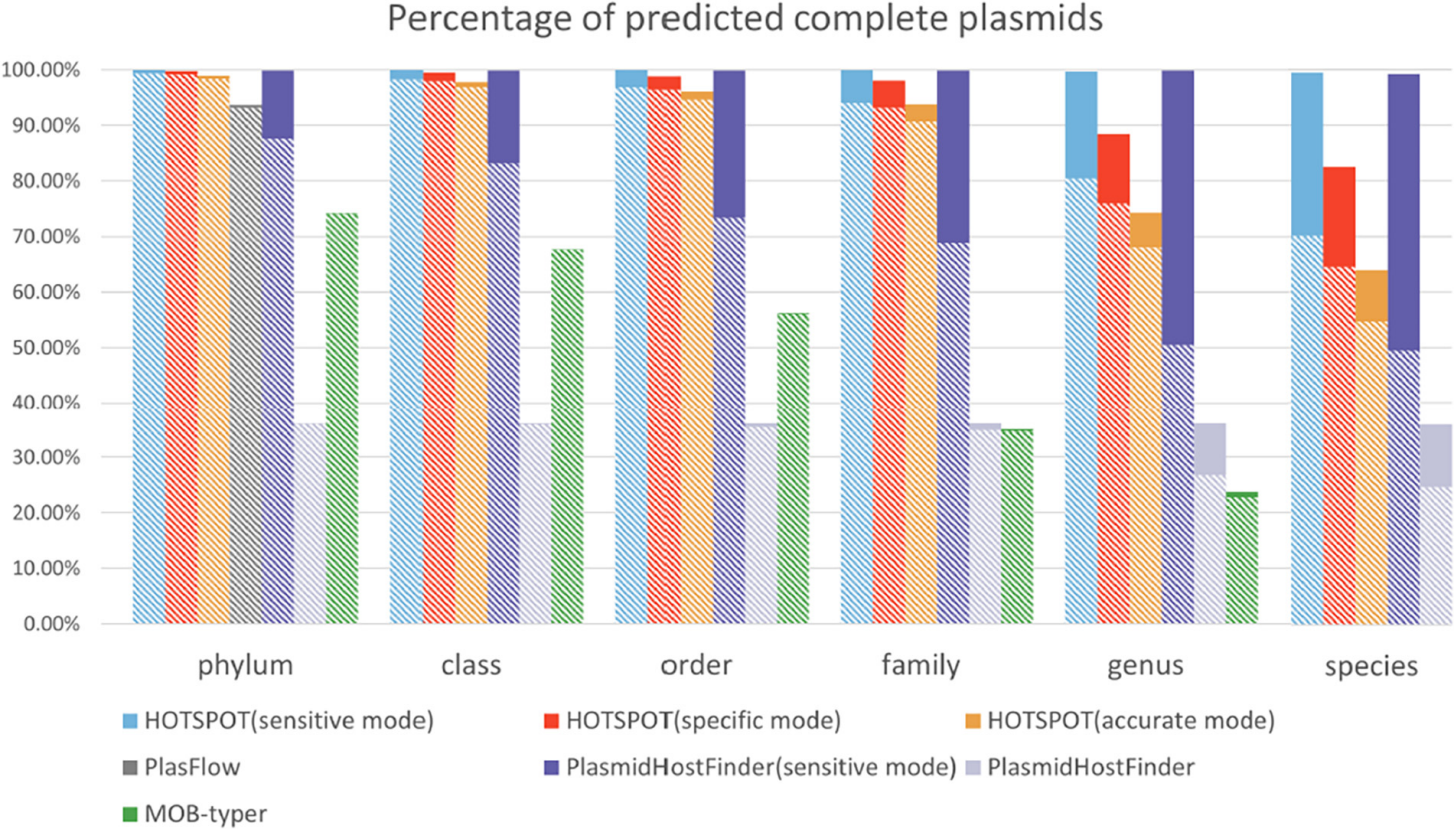
The prediction performance of each tool at different ranks from phylum to species on the complete plasmid test set. Each bar shows the percentage of predicted plasmids (prediction rate). The solid part of the bar reflects the error rate, and the patterned part at the bottom shows the accuracy. Notably, PlasFlow assigns phylum-level labels to plasmid hosts, thus only having the performance evaluation for phylum.

We compared the performance of HOTSPOT (pre-trained models described in Section 2.4) with the other three tools on the complete plasmids. Figure 4 shows the benchmark results on the complete plasmid test set. Each bar’s height reflects the prediction rate. Within the bar, the solid part represents the error rate, and the patterned part represents the accuracy. We can observe that HOTSPOT outperforms other approaches across different taxonomic ranks. More specifically, although the performance of all tools decreases from phylum to species, HOTSPOT can still maintain a high prediction rate and accuracy. While comparing the performance of HOTSPOT’s three modes (described in Section 2.3), it shows that the sensitive mode can achieve the best prediction rate but the lowest accuracy, which is the opposite of the accurate mode. The specific mode strikes a balance between the prediction rate and accuracy. Therefore, when enabling the early stop mechanism, HOTSPOT will sacrifice some prediction rate for higher accuracy. In the following experiments, we evaluate all tools’ performance with F1-score and employ sensitive mode for HOTSPOT and PlasmidHostFinder.

**Figure 5.**
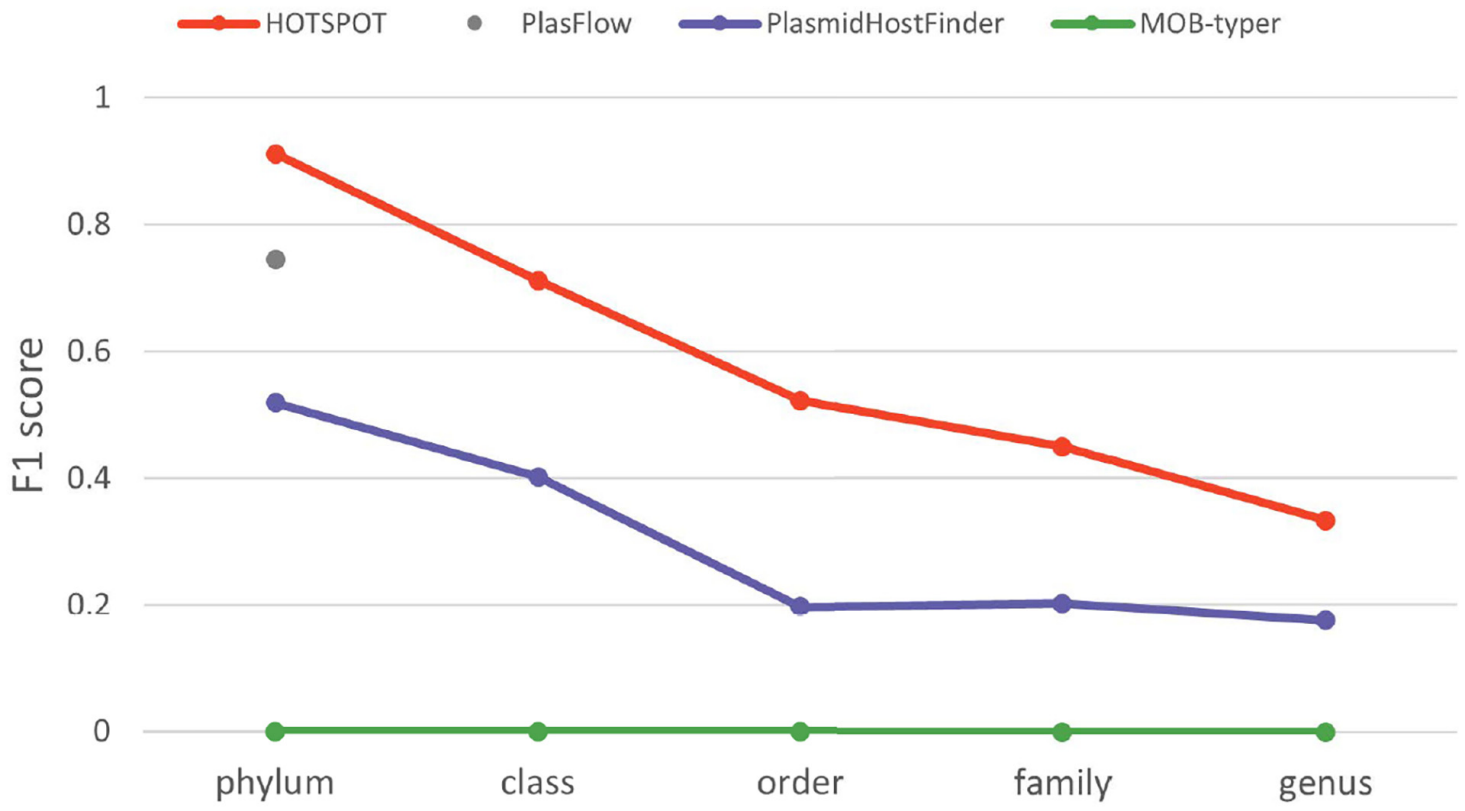
The prediction performance on the first group of the complete plasmids with low similarity to the training set. In this group, all the 102 plasmids’ maximum Mash similarities against the training set are 0%. *X*-axis: the five taxonomic ranks. *Y*-axis: the F1-score.

In addition, to show how the train-versus-test similarity affects plasmid host prediction, we compare each tool’s performance on four groups of complete plasmids with different maximum similarities with the training data. The details can be found in Supplementary Section S8.2. Figure 5 shows the benchmark results on plasmids in the first group, whose maximum Mash similarities against the training set are 0%, indicating these plasmids do not share any 21-mer with the 21 212 training plasmids. As shown in Fig. 5, HOTSPOT outperforms the other three tools on this plasmid group, suggesting that HOTSPOT is more reliable on plasmids with low similarity to the training data. Across all taxonomic ranks, MOB-typer does not have any output because it predicts plasmid hosts via Mash-based clustering and thus cannot work on plasmids with Mash similarities of 0% to plasmids in its database.

As described in Section 2.1, HOTSPOT’s taxonomic label set is smaller than the complete bacterial taxa, especially at lower ranks like genus and species. For a novel plasmid whose host label (e.g. species) is not part of the training set, HOTSPOT can return the taxa with higher ranks. In Supplementary Section S8.1, we conducted host prediction for 162 and 734 complete plasmids in the test set, whose genus-level and species-level labels are not in our label set, respectively.

### 3.3 Experiments on the short contigs

Table 2 shows the benchmark results on the short plasmid contigs from the test set at the family, genus, and species level (the complete results from phylum to species are shown in Supplementary Section S9). The four columns correspond to the performance on contigs with different lengths: 1.5, 3, 5, and 10 kb. PlasmidHostFinder trained multiple models for different contig lengths, making the testing tedious. We choose the best model that can be obtained with reasonable efforts. For example, we used the default 1.5 kb pre-trained model on 1.5 kb contigs and applied the complete plasmid model of PlasmidHostFinder on the 5 and 10 kb contigs. Table 2 shows that the performance of all pipelines increases with the increase of the contig length. This is expected because longer sequences carry more valuable features for host prediction, including proteins and *k*-mers. Even so, HOTSPOT still performs best on input contigs with various lengths. Among all experiments, MOB-typer tends to achieve a low prediction rate but very high accuracy.

**Table 2.** The F1-score of each tool from the family to species level on the short plasmid contigs.

Rank	Tools	1.5 kb	3 kb	5 kb	10 kb
	HOTSPOT	0.8497	0.8732	0.8836	0.9025
Family	PlasmidHostFinder	0.6861	0.6767	0.6732	0.6716
	MOB-typer	0.1474	0.2104	0.2601	0.3757
	HOTSPOT	0.6803	0.7114	0.7344	0.7557
Genus	PlasmidHostFinder	0.5101	0.5023	0.5025	0.4955
	MOB-typer	0.0739	0.1199	0.1563	0.2272
Species	HOTSPOT	0.5927	0.6199	0.6447	0.6597
	PlasmidHostFinder	0.4956	0.4922	0.4942	0.492

*Note*: The four columns show the F1-score on contigs of 1.5, 3, 5, and 10 kb, respectively.

### 3.4 Experiments on the mock metagenomic datasets

**Figure 6.**
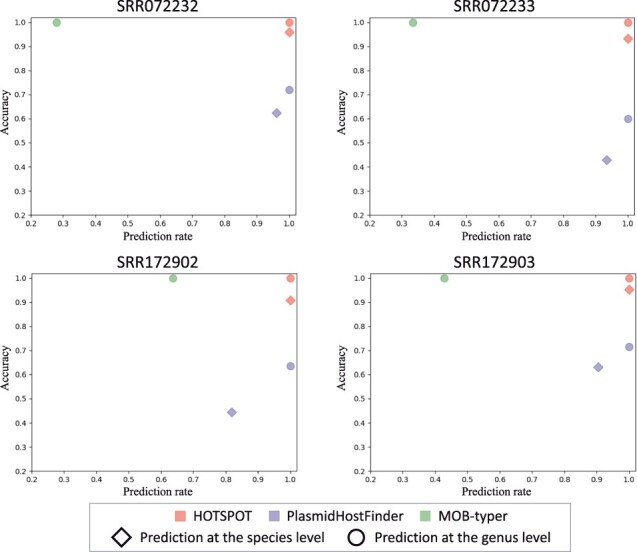
The prediction performance on the four mock metagenomic datasets. *X*-axis: Prediction rate. *Y*-axis: Accuracy. Each rhombus represents a genus-level prediction, and each circle represents a species-level prediction.

We compare the three tools on four real shotgun-sequenced metagenomic data from the HMP Metagenomic WGS Projects. The assembled contigs identified as plasmids are used as input. The prediction results of all methods are shown in Fig. 6, where *X* and *Y*-axis represent prediction rate and accuracy, respectively. Thus, point (1,1) refers to the best performance. Furthermore, the area of the rectangle formed by the prediction point and the origin (Prediction rate×Accuracy) equals the proportion of correctly predicted contigs in the total input plasmid contigs. Generally, the rectangle’s areas formed by the other tools are smaller than HOTSPOT at the genus and species level. A closer look shows that HOTSPOT achieves an average species-level accuracy of 93.87% and an average genus-level accuracy of 100% for the four datasets. This experiment reveals that HOTSPOT can be generalized to real metagenomic data.

### 3.5 Experiments on the Hi-C dataset

**Figure 7.**
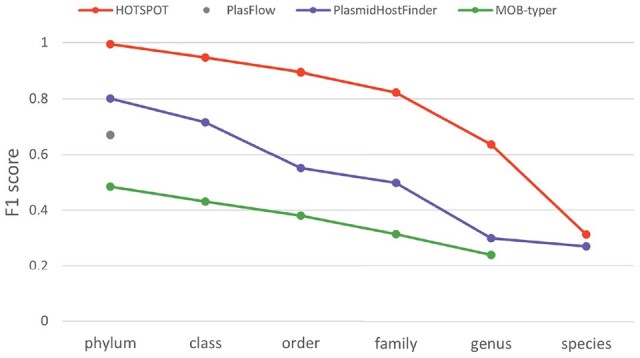
The prediction performance on the Hi-C dataset. *X*-axis: the six taxonomic ranks. *Y*-axis: the F1-score.

The Hi-C data provides another way of obtaining the linkage between plasmids and their hosts. It can be applied to underrepresented environmental samples and provide host information for highly diverged plasmids. This dataset poses a hard case for host prediction at the species level. As shown in Fig. 7, HOTSPOT’s F1-score drops considerably at the species level. Nevertheless, HOTSPOT achieves good prediction performance above the genus level on the Hi-C dataset. In addition, we also compare HOTSPOT’s results on the identified plasmid contigs with the taxa classified by the standard metagenomic classifier, Kraken 2 (Wood et al. 2019), on the raw Hi-C reads. Ideally, the predicted hosts’ taxa should be the subset of the taxa classified by Kraken 2 because not all bacteria in the sample carry plasmids. The comparison results can be found in Supplementary Section S11.

## 4 Discussion

In this work, we introduce a new plasmid host prediction tool, HOTSPOT. It achieves higher accuracy than available tools by organizing Transformer-based classifiers in a phylogenetic tree. The state-of-the-art language model Transformer allows HOTSPOT to automatically learn the importance of proteins and their associations, leading to higher recall than alignment-based tools. The tree organization allows it to conduct hierarchical host prediction from phylum to species. Together with early stop, HOTSPOT can better accommodate the challenge of incomplete label set and training data than available machine learning-based tools. Our design is validated by the experimental results on multiple types of data.

Nevertheless, we have several goals to optimize the host prediction in the future. First, the functional annotation of plasmid proteins may greatly improve the understanding of the host ranges. Second, the accuracy of HOTSPOT suffers from the different distribution between our training data and real metagenomic data. Recently, many taxonomic classification methods have achieved promising performance on assembled bacterial chromosomes from metagenomic data. Thus, the bacterial taxa produced by these tools can reduce the search space of HOTSPOT, further improving the prediction accuracy. Finally, predicting plasmid contigs from metagenomic data is still challenging, which can introduce false positives to HOTSPOT’s input. To minimize the false positives, we recommend a pipe of plasmid identification with high precision (see Supplementary Section S6). Yet, this pipeline relies on alignment-based plasmid identification and thus can miss some highly diverged plasmids. HOTSPOT can benefit from improved plasmid identification methods.

## Supplementary Material

btad283_Supplementary_Data
