# Supplementary material for: HOTSPOT: hierarchical host prediction for assembled plasmid contigs with transformer
Source: Bioinformatics. 2023 Apr 22;39(5):btad283. doi: 10.1093/bioinformatics/btad283 (PMC10159655; doi:10.1093/bioinformatics/btad283)
Supplement: btad283_Supplementary_Data [file btad283_supplementary_data.pdf]

Supplementary information for "HOTSPOT: Hierarchical hOst predicTion of aSsembled Plasmid cOntigs with Transformer"

Yongxin Ji, Jiayu Shang, Xubo Tang, and Yanni Sun

Electrical Engineering Department, City University of Hong Kong, Kowloon, Hong Kong SAR

Mar 2023

1 Overview of the MOB/MPF and Inc databases

The MOB/MPF and Inc identification databases are combined from a review article [1] and several plasmid typing tools, including MOBscan [2], PlasmidFinder, and pMLST [3]. As a result, the integrated databases consist of 9 MOB types, 4 MPF types, and 22 Inc groups. All these typing markers are from prior experimental evidence. In addition, the MOB/MPF proteins will not exist on non-mobilizable plasmids. Thus, the three typing methods only work for specific plasmids. We used BLAST on the curated 30,284 plasmids to record the percentage of identified plasmids by MOB, MPF, and Inc typing schemes. In Figure 1, the box plot shows the percentages of identified plasmids hosted by 105 different families, indicating that the MOB proteins can cover the broadest taxonomic range, and the Inc groups can only match plasmids of limited taxonomic units. For example, plasmids from 59 families out of 105 cannot be detected by the Inc database, and the family *Listeriaceae* achieves the best match with the Inc groups, where 79 plasmids out of 93 (about 85%) have one or more identified Inc groups.

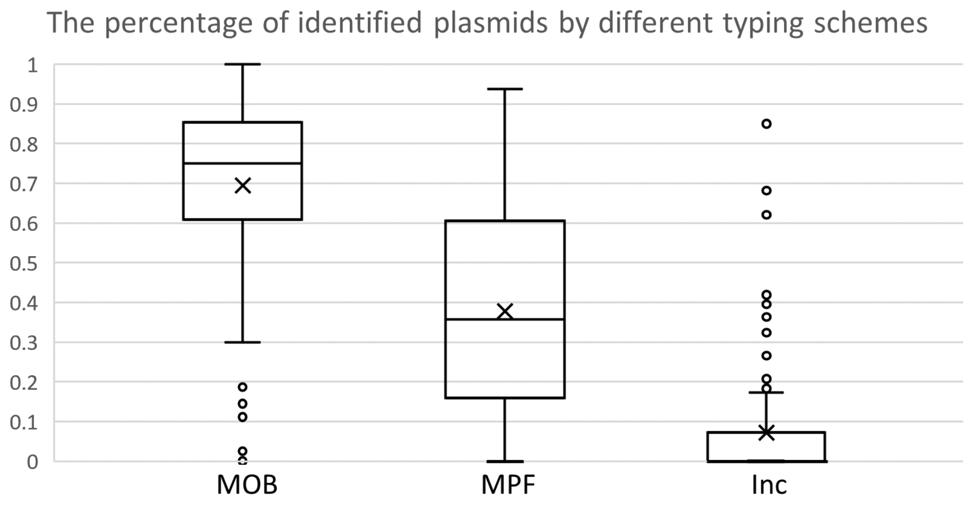

Figure 1: The box plot used to display the distribution of identified plasmid (MOB, MPF, and Inc) percentages hosted by 105 families. The three quartiles are plotted by lines. In particular, the cross represents the mean value, and the small circle represents an outlier.

2 Performance assessment of Prodigal

Dimonaco *et al.* showed that Prodigal is one of the most comprehensive prokaryotic gene prediction tools through benchmark experiments using 12 informative metrics (M1 to M12) on multiple high-quality datasets [4]. In particular,

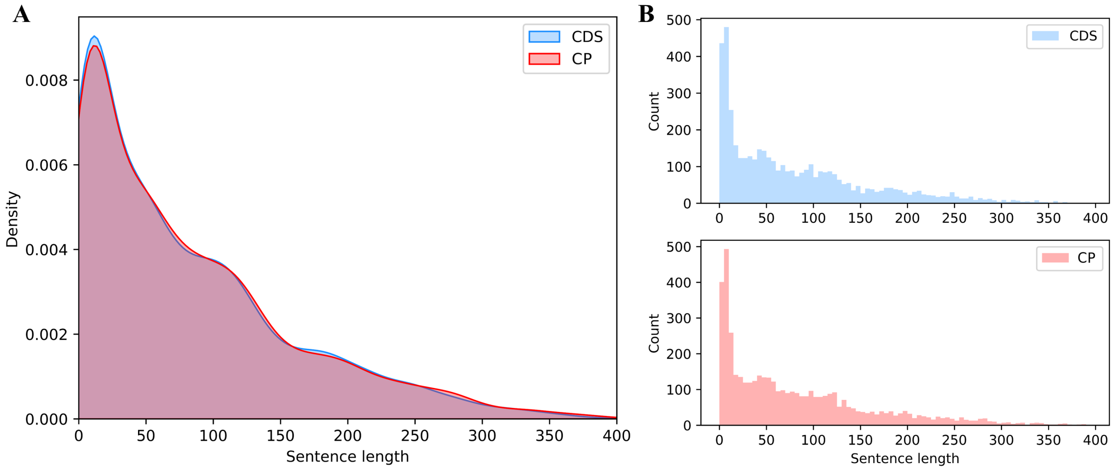

Figure 2: (A) The continuous probability density distribution of two groups of PC sentence lengths. The blue shade with the legend 'CDS' represents the PC sentences built with annotated proteins in NCBI, and the red shade with the legend 'CP' represents the PC sentences built with Prodigal's CPs. (B) The histograms representing the distributions of the two groups of PC sentence lengths. The total count is 4,518, and the bin size is 5.

Prodigal was ranked top on the M2 metric, percentage of predicted Coding Sequences (CDSs) that detected a gene, indicating Prodigal's false positive rate competes favorably with other tools. Despite the promising results, we will further examine Prodigal's false positive rate and its influence on HOTSPOT in this section.

We first downloaded available protein sequences translated from annotated CDSs in the NCBI RefSeq database of the 4,536 complete plasmids in the test set. 4,518 out of 4,536 plasmids have annotated protein sequences. Focusing on the 4,518 plasmids, we used Diamond BLASTP [5] to compare Prodigal's 365,709 computationally predicted proteins (CPs) against the 358,804 annotated proteins in NCBI. The results show that 329,943 out of 365,709 CPs (~90.22%) have reliable alignments with the annotated proteins (E-value < 0.001 [6]) on the same plasmids. Thus, Prodigal achieved an acceptable false positive rate of 9.78% in this task.

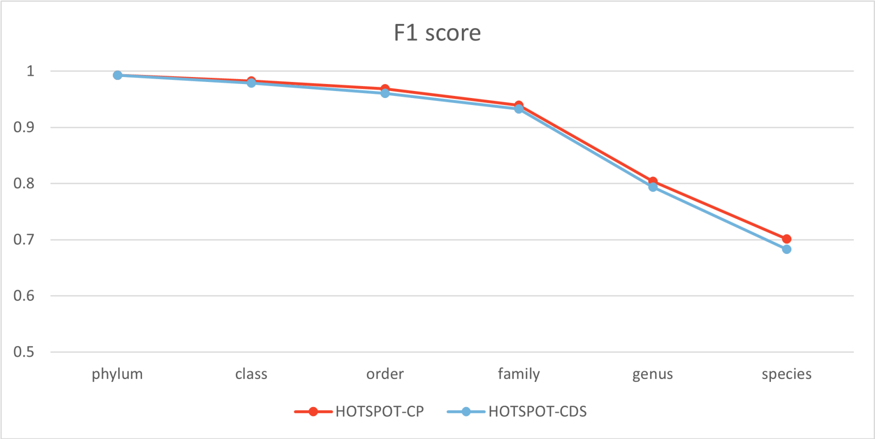

Figure 3: The prediction performance on the 4,518 plasmids in the test set, which have annotated proteins in NCBI. X-axis: the six taxonomic ranks. Y-axis: the F1 score. The red line represents the performance of standard HOTSPOT (using CPs as features), and the blue line represents the performance of HOTSPOT using annotated proteins.

Second, some false positives will be removed when building PC sentences because they cannot be aligned to any plasmid protein in our database. Thus, we constructed PC sentences for the 4,518 plasmids using the annotated proteins in NCBI and compared their lengths with those built with CPs. As shown in Figure 2, the length distributions

of the two groups of sentences are nearly consistent.

Finally, we took the two groups of PC sentences combined with corresponding MOB/MPF sentences and Inc one-hot vector as inputs for HOTSPOT and compared their performance on host prediction for the 4,518 plasmids. The benchmark results are shown in Figure 3. No significant difference can be observed between the performance of using the two groups of sentences. Besides, the F1 score of HOTSPOT with predicted proteins is even slightly higher. Thus, we can conclude that Prodigal is reliable in generating protein features for predicting plasmid hosts with HOTSPOT.

3 Analysis of the alignment quality

There are two types of alignments in this work: DNA-DNA alignments conducted by BLASTN and protein-protein conducted by DIAMOND BLASTP. Thus, we will analyze the quality of each type of alignment in this section.

3.1 DNA-DNA alignments for Inc group assignment

The Inc group assignment is realized by aligning query plasmid contigs to the conserved replicon sequences (probes) using BLASTN. The replicons in the database are mainly integrated from the tools PlasmidFinder and pMLST [3]. Thus, we implement the alignment filtering consistent with PlasmidFinder, using the >80% nucleotide identity and 96% subject coverage criteria. In this manner, most multiple alignments on different Inc groups will be avoided, making the Inc one-hot vector construction more precise.

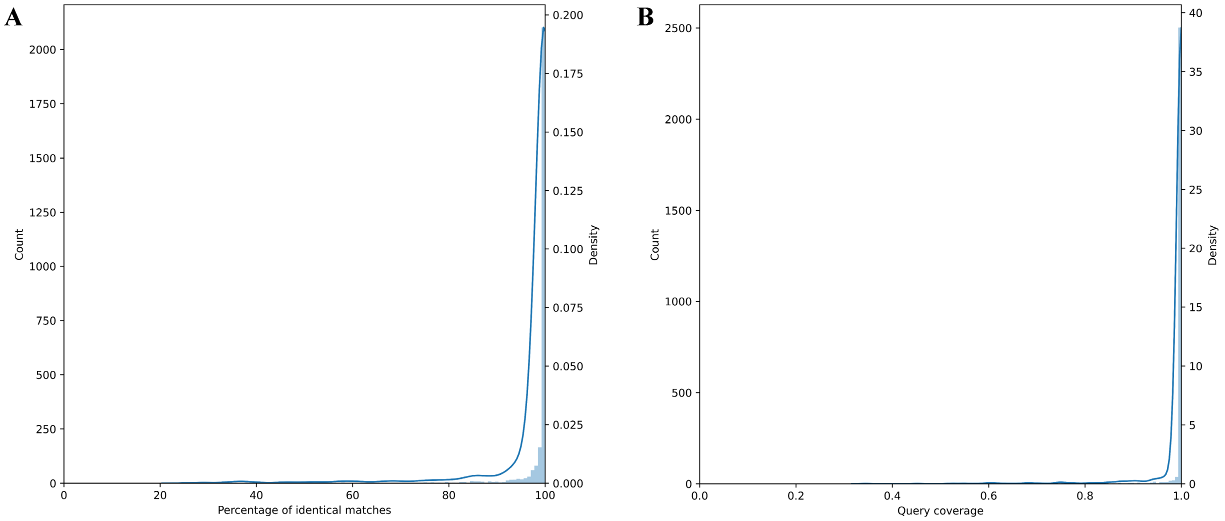

Figure 4: (A) The distribution of the percent identity of the 2730 protein-protein alignments. The distribution is visualized using both the continuous probability density curve (the right Y-axis) and the histogram with bin size 1 (the left Y-axis). (B) The distribution of the query coverage of the 2730 protein-protein alignments, whose representation style is the same as Figure 4A.

3.2 protein-protein alignments for sentence construction

The first step for constructing PC and MOB/MPF sentences is to align predicted proteins to the curated protein databases using DIAMOND BLASTP. As introduced in [6], protein-protein alignment statistics are more accurate than DNA-DNA alignment statistics. Therefore, we keep the alignments with the maximum E-value 1e-3 ($E \leq 0.001$) for both the MCL database building and sentence encoding (see Methods 2.2.1).

Then, we will take the 501 plasmid contigs identified from the Hi-C dataset to illustrate the quality of the protein-protein alignments. In total, there are 2,730 alignments between the predicted proteins and their best-matching proteins in the MCL database. The percent identity and query coverage distributions of the 2,730 alignments are presented in Figure 4. We can observe that most of the alignments have both high percent identity and query coverage.

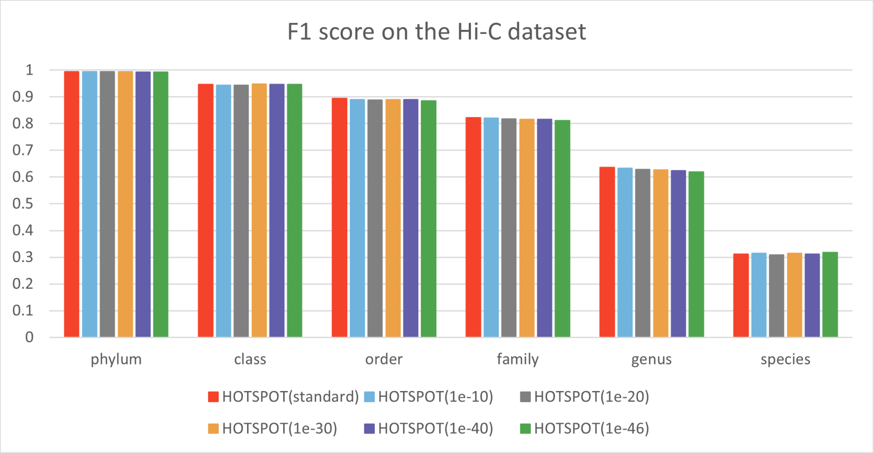

Figure 5: The F1 score of HOTSPOT using different E-value cutoffs on the six ranks.

We also use six different E-values, 1e-3 (standard), 1e-10, 1e-20, 1e-30, 1e-40, and 1e-46, for performance comparison of plasmid host prediction on the Hi-C dataset. We choose the smallest cutoff of 1e-46 for comparison because some plasmids have no alignment and prediction with a cutoff smaller than 1e-47. As shown in Figure 5, HOTSPOT with six different E-value cutoffs nearly achieves the same performance, proving that using 1e-3 as the default E-value for protein-protein alignments is reasonable.

4 Architecture of the Transformer model

4.1 The embedding layers

The token is like a word in NLP applications. Thus, we have to employ the embedding algorithm to convert each token into a vector. Because Transformer does not involve convolution and recurrence, the recorded positional index vector also flows through an embedding layer to encode the position information. As shown in Figure 2B (in the main text), a Transformer block includes two embedding layers: token embedding and positional embedding. Traditional encoding methods like one-hot cannot handle the tremendous amount of PC tokens (108,274), leading to a very sparse vector. Hence for each embedding layer, we implement a fully connected layer (FC layer), which can translate the numerous tokens into a relatively low-dimensional space. In this manner, the embedding layer is a lookup table, where the inputs are indexes (the token IDs), and the extracted results are fixed-length vectors of defined size. Due to the same dimension of the resultant vectors generated by the two embedding layers, we will sum them together for feeding the Transformer block afterward. Specifically, the embedding layer can be mathematically defined as:

$$\begin{cases} \widetilde{I_s} = FC(I_s, W_s) \\ \widetilde{I_p} = FC(I_p, W_p) \\ X = \widetilde{I_s} + \widetilde{I_p} \end{cases} \tag{1}$$

where I_s is the input sentence and I_p is the positional index vector. $\widetilde{I_s}$ and $\widetilde{I_p}$ are the resulting embedded vectors of I_s and I_p, respectively. X is the final output. The dimensions of W_s and W_p are $N \times embed$ and $len \times embed$, respectively, where N is the number of tokens, len is the sentence length, and $embed$ is the dimension of the embedded vectors. Because the values of $embed$ for $\widetilde{I_s}$ and $\widetilde{I_p}$ are the same, we can sum them as the final output. Besides, in the two Transformers, it is different for the values of the parameters $(N, len, embed)$, which are preset to (108,274, 400, 256) in the PC Transformer and (13, 50, 32) in the MOB/MPF Transformer. Using these supervised neural networks for embedding, the tokens with similar categories, such as replication initiator proteins (Reps) of the same type, will be closer to each other in the embedding space. The padding token with zero ID will not participate in the embedding computation.

4.2 The self-attention layer

Each token will be converted through the embedding layers into a vector with length *embed*. Subsequently, the embedded matrix with dimension $len \times embed$ will be fed into the self-attention layer. Using three FC layers, three matrices, a Query matrix (Q), a Key matrix (K), and a Value matrix (V) are created from the embedded matrix:

$$\begin{cases} Q = FC(X, W^Q) \\ K = FC(X, W^K) \\ V = FC(X, W^V) \end{cases} \tag{2}$$

where W^Q, W^K, and W^V are three projection matrices to be trained with dimension $embed \times d_{head}$. The value of d_{head} is determined by the multi-head attention, which will be introduced later. The query/key/value functions are similar to the retrieval system applications. Taking the PC Transformer as an example, we will map the query (translated proteins) against several keys (specific proteins and their relative positions) to acquire the host's best-matched taxon (values). Following this idea, we can realize the host prediction by:

$$Attention(Q, K, V) = Softmax(\frac{Q \times K^T}{\sqrt{d_k}})V \tag{3}$$

As shown in Equation 3, an attention score matrix will be first computed to learn the associations between tokens by multiplying Q and K. The attention score matrix is a square matrix with dimension $len \times len$. The higher value in the matrix represents the stronger association. Then, we employ the Softmax function to generate normalized scores, which act as the weights for the weighted average of values (V). It is worth noting that the scores have to be divided by a scaling factor $\sqrt{d_k}$ to enable more stable gradients when training. By default, we set $d_k = embed = 256$.

4.3 Feed-forward networks

The output of the self-attention layer will be fed into the 2-layer feed-forward networks. The residual connection [7] and layer normalization [8] are employed for the outputs of both the self-attention layer and the feed-forward networks (Figure 2B in the main text). As a result, we can effectively handle the gradient vanishing and overfitting problem. Then, the feature vectors output by the two Transformer blocks and the encoded Inc one-hot vector will be concatenated and flow through an FC layer for the final multi-class taxon classification. The final output normalized by the Softmax function can reflect the probability of the input plasmid contig's host belonging to each taxon.

5 Duplication removal for downloaded plasmids sequences

To remove redundant plasmids, we first used Dashing [9] with $k = 31$ to calculate pairwise k-mer-based Jaccard similarities for all the plasmid genomes. Then, we implemented complete-linkage hierarchical clustering based on the similarity matrix to group plasmids into a dendrogram. Finally, we chose a similarity cutoff of 95% to cut the dendrogram into 30,284 clusters, where the Jaccard similarity between every two plasmids in the same cluster is greater than 95%. Notably, the 95% k-mer-based Jaccard similarity is roughly equal to 99.89% average nucleotide identity (ANI) similarity [10], indicating that all plasmids in a cluster are highly similar. Therefore, we randomly select one representative plasmid from each cluster, which results in 30,284 plasmids for the model training.

6 Analysis of HOTSPOT with noisy inputs

6.1 Performance of alignment-based plasmid identification tools

For higher precision of plasmid identification, we recommend that users use alignment-based tools, including MOB-recon [11] and Platon [12]. We use the simulated MAG dataset to illustrate the performance of these two tools. The dataset contains 10,395 assembled contigs, where 10,221 are chromosome-borne, and the other 174 are plasmid-borne. We run both MOB-recon and Platon with default parameters on the 10,395 contigs. As a result, MOB-recon identifies 119 contigs as plasmids, and 7 of them are false positives. Platon identifies 155 contigs as plasmids, and 26 are false positives. However, if we use the intersection of the results of the two tools, we can successfully get 91

identified plasmids with 0 false positive (100% precision and 52.3% recall). In conclusion, using the intersection of MOB-recon's and Platon's output can achieve high precision in plasmid identification.

6.2 The Behavior of HOTSPOT on chromosome-borne contigs

Because identifying plasmids from metagenomic data requires a separate set of efforts, HOTSPOT is not designed or optimized for this task. When a non-plasmid is input to HOTSPOT, two situations can happen. First, if the input does not contain any proteins homologous to our protein clusters, the sentence cannot be built, and thus the input will be rejected. Second, if the input can produce matches to our protein clusters, a sentence will be formed and lead to a prediction. As many chromosomes can share homologous proteins with plasmids, the second situation is not rare. In this case, we should evaluate whether HOTSPOT can return its correct taxonomic label. In this experiment, we conduct this experiment and report the taxonomic classification performance of using HOTSPOT on chromosome contigs. We first run HOTSPOT on the 33 false positives by MOB-recon and Platon. For comparison, we also input 200 randomly selected contigs from the 10,221 chromosome-borne contigs into HOTSPOT. 5 of the 200 chromosome-borne contigs cannot be encoded into PC sentences (no similar protein to our database) and thus have no prediction. Each input contig is labeled with the taxonomic lineage of its mapped chromosomes. The performance of the two groups of contigs is shown in Figure 6. We can observe that HOTSPOT performs better on the 33 false positives. The reason should be that the false positives generated by alignment-based tools usually have regions of local similarity or homologous proteins with the plasmids in their databases. HOTSPOT can thus make better taxonomic classification because of the sequence similarities. In addition, some persistent plasmids can be fixed in their host chromosomes through the transition process [13].

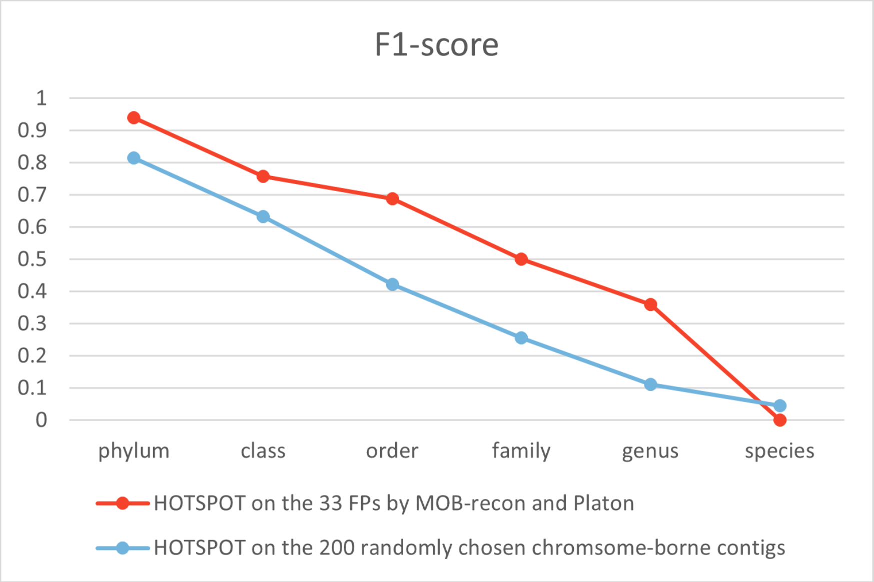

Figure 6: The taxonomic classification performance of HOTSPOT (sensitive mode) on the 33 false positives and the 200 chromosome contigs.

7 The reference genome lists of the MAG and mock datasets

This section lists all the reference genomes in the MAG and mock datasets. In particular, the reference plasmids existing in our training set are labeled with a star '*' in the tables below. When comparing the performance, the contigs mapped to the starred reference plasmids will be removed first.

7.1 The reference genomes to simulate the MAG dataset

The selected species and source code to simulate the MAG dataset can be found at https://github.com/fmaguire/MAG_gi_plasmid_analysis [14]. 26 out of 65 reference plasmids are in our training set and starred.

Organism	BioSample	Accession	Seq_type
Mycobacterium gilvum	SAMN02598346	NC_009338	chromosome
Mycobacterium gilvum	SAMN02598346	NC_009339*	plasmid
Mycobacterium gilvum	SAMN02598346	NC_009340*	plasmid
Mycobacterium gilvum	SAMN02598346	NC_009341*	plasmid
Shigella dysenteriae	SAMN02603600	NC_007606	chromosome
Shigella dysenteriae	SAMN02603600	NC_009344	plasmid
Shigella dysenteriae	SAMN02603600	NC_007607	plasmid
Shigella flexneri	SAMN02603599	NC_004337	chromosome
Shigella flexneri	SAMN02603599	NC_004851	plasmid
Campylobacter subantarcticus	SAMN03248548	NZ_CP007772	chromosome
Pseudomonas syringae group genomosp. 3	SAMN02604017	NC_004578	chromosome
Pseudomonas syringae group genomosp. 3	SAMN02604017	NC_004633*	plasmid
Pseudomonas syringae group genomosp. 3	SAMN02604017	NC_004632*	plasmid
Bordetella petrii	SAMEA3138272	NC_010170	chromosome
Streptococcus macedonicus	SAMEA2272145	NC_016749	chromosome
Streptococcus macedonicus	SAMEA2272145	NC_016750*	plasmid
Lactobacillus fermentum	SAMD00060917	NC_010610	chromosome
Streptococcus constellatus	SAMN02603657	NC_022236	chromosome
Acetobacter pasteurianus	SAMN06855882	NZ_CP022374	chromosome
Synechococcus sp. PCC 8807	SAMN04422305	NZ_CP016483	chromosome
Synechococcus sp. PCC 8807	SAMN04422305	NZ_CP016484	plasmid
Synechococcus sp. PCC 8807	SAMN04422305	NZ_CP016485	plasmid
Synechococcus sp. PCC 8807	SAMN04422305	NZ_CP016486	plasmid
Synechococcus sp. PCC 8807	SAMN04422305	NZ_CP016487	plasmid
Synechococcus sp. PCC 8807	SAMN04422305	NZ_CP016488	plasmid
Synechococcus sp. PCC 8807	SAMN04422305	NZ_CP016489	plasmid
Erwinia sp. Ejp617	SAMN02603467	NC_017445	chromosome
Erwinia sp. Ejp617	SAMN02603467	NC_017442*	plasmid
Erwinia sp. Ejp617	SAMN02603467	NC_017446*	plasmid
Erwinia sp. Ejp617	SAMN02603467	NC_017443*	plasmid
Erwinia sp. Ejp617	SAMN02603467	NC_017447*	plasmid
Erwinia sp. Ejp617	SAMN02603467	NC_017444*	plasmid
Bacillus thuringiensis	SAMN02598265	NC_005957	chromosome
Bacillus thuringiensis	SAMN02598265	NC_006578	plasmid
Acinetobacter soli	SAMN05554081	NZ_CP016896	chromosome
Acinetobacter soli	SAMN05554081	NZ_CP016897	plasmid
Acinetobacter soli	SAMN05554081	NZ_CP016898	plasmid
Acinetobacter soli	SAMN05554081	NZ_CP016899	plasmid
Acinetobacter soli	SAMN05554081	NZ_CP016900	plasmid
Acinetobacter soli	SAMN05554081	NZ_CP016901	plasmid
Acinetobacter soli	SAMN05554081	NZ_CP016902	plasmid
Acinetobacter soli	SAMN05554081	NZ_CP016903	plasmid
Lactobacillus paracollinoides	SAMN04505735	NZ_CP014912	chromosome
Lactobacillus paracollinoides	SAMN04505735	NZ_CP014913*	plasmid
Lactobacillus paracollinoides	SAMN04505735	NZ_CP014914*	plasmid
Moraxella osloensis	SAMN04435858	NZ_CP014234	chromosome

Moraxella osloensis	SAMN04435858	NZ_CP014238	plasmid
Moraxella osloensis	SAMN04435858	NZ_CP014237	plasmid
Moraxella osloensis	SAMN04435858	NZ_CP014236	plasmid
Moraxella osloensis	SAMN04435858	NZ_CP014235	plasmid
Lactobacillus backii	SAMN04505726	NZ_CP014623	chromosome
Lactobacillus backii	SAMN04505726	NZ_CP014624	plasmid
Lactobacillus backii	SAMN04505726	NZ_CP014625	plasmid
Lactobacillus backii	SAMN04505726	NZ_CP014626	plasmid
Lactobacillus backii	SAMN04505726	NZ_CP014627	plasmid
Lactobacillus backii	SAMN04505726	NZ_CP014628	plasmid
Lactobacillus backii	SAMN04505726	NZ_CP014629	plasmid
Lactobacillus backii	SAMN04505726	NZ_CP014630	plasmid
Lactobacillus backii	SAMN04505726	NZ_CP014631	plasmid
Lactobacillus backii	SAMN04505726	NZ_CP014632	plasmid
Lactobacillus backii	SAMN04505726	NZ_CP014633	plasmid
Klebsiella oxytoca	SAMN02786843	NZ_CP008788	chromosome
Klebsiella oxytoca	SAMN02786843	NZ_CP008789*	plasmid
Klebsiella oxytoca	SAMN02786843	NZ_CP008790*	plasmid
Klebsiella oxytoca	SAMN02786843	NZ_CP008791*	plasmid
Methylobacterium nodulans	SAMN00000043	NC_011894	chromosome
Methylobacterium nodulans	SAMN00000043	NC_011892	plasmid
Methylobacterium nodulans	SAMN00000043	NC_011887	plasmid
Methylobacterium nodulans	SAMN00000043	NC_011893*	plasmid
Methylobacterium nodulans	SAMN00000043	NC_011895*	plasmid
Methylobacterium nodulans	SAMN00000043	NC_011888*	plasmid
Methylobacterium nodulans	SAMN00000043	NC_011889*	plasmid
Methylobacterium nodulans	SAMN00000043	NC_011890*	plasmid
Pelagibaca abyssi	SAMN04562614	NZ_CP015093	chromosome
Pelagibaca abyssi	SAMN04562614	NZ_CP015091*	plasmid
Pelagibaca abyssi	SAMN04562614	NZ_CP015090	plasmid
Pelagibaca abyssi	SAMN04562614	NZ_CP015092*	plasmid
Pelagibaca abyssi	SAMN04562614	NZ_CP015095	plasmid
Pelagibaca abyssi	SAMN04562614	NZ_CP015089	plasmid
Pelagibaca abyssi	SAMN04562614	NZ_CP015094*	plasmid
Pelagibaca abyssi	SAMN04562614	NZ_CP015097	plasmid
Pelagibaca abyssi	SAMN04562614	NZ_CP015096*	plasmid
Paenibacillus bovis	SAMN04193493	NZ_CP013023	chromosome
Paenibacillus bovis	SAMN04193493	NZ_CP021170	plasmid
Caulobacter segnis	SAMN02598513	NC_014100	chromosome
Pedobacter steynii	SAMN05582931	NZ_CP017141	chromosome
Actinomyces sp. Marseille-P2985	SAMEA4555081	NZ_LT635457	chromosome
Streptococcus sp. I-G2	SAMN02299294	NC_022584	chromosome
Streptomyces parvulus	SAMN04924834	NZ_CP015866	chromosome
Streptomyces parvulus	SAMN04924834	NZ_CP015867	plasmid
Chlamydia pneumoniae	SAMN02602979	NC_005043	chromosome
Lactobacillus acidophilus	SAMN02603047	NC_006814	chromosome
Clostridium baratii	SAMN03222823	NZ_CP006905	chromosome
Clostridium baratii	SAMN03222823	NZ_CP006906*	plasmid
Akkermansia muciniphila	SAMN00138213	NC_010655	chromosome

7.2 The reference plasmids identified in the four mock datasets

The microorganisms in the four mock metagenomes were identified using genome-specific markers by the authors in [15], and the results can be found in [15]'s Supplementary Table S1. Based on the identified strain names, we downloaded all available plasmids from NCBI, which are listed in the table below. 16 out of 50 reference plasmids are in our training set and starred.

Accession	Organism	Accession	Organism
NC_005008	Staphylococcus epidermidis	NZ_AKBU01000008	Cereibacter sphaeroides
NC_005007	Staphylococcus epidermidis	NZ_AKVW01000003	Cereibacter sphaeroides
NC_005006*	Staphylococcus epidermidis	NZ_AKVW01000004	Cereibacter sphaeroides
NC_005005*	Staphylococcus epidermidis	NZ_AKVW01000005	Cereibacter sphaeroides
NC_005004*	Staphylococcus epidermidis	NZ_AKVW01000006	Cereibacter sphaeroides
NC_005003	Staphylococcus epidermidis	NZ_AKVW01000007	Cereibacter sphaeroides
NC_005707*	Bacillus cereus	NZ_CP015084	Deinococcus radiodurans
NC_000959*	Deinococcus radiodurans	NZ_CP015083	Deinococcus radiodurans
NC_000958	Deinococcus radiodurans	NZ_CP030273	Cereibacter sphaeroides
NC_009007*	Cereibacter sphaeroides	NZ_CP030274	Cereibacter sphaeroides
NC_007488	Cereibacter sphaeroides	NZ_CP030275	Cereibacter sphaeroides
NC_007489*	Cereibacter sphaeroides	NZ_CP030276	Cereibacter sphaeroides
NC_007490*	Cereibacter sphaeroides	NZ_CP043846	Staphylococcus epidermidis
NC_009008*	Cereibacter sphaeroides	NZ_CP049364	Acinetobacter baumannii
NC_009083	Acinetobacter baumannii	NZ_CP049365*	Acinetobacter baumannii
NC_009084	Acinetobacter baumannii	NZ_CP053099*	Acinetobacter baumannii
NC_010066*	Staphylococcus aureus	NZ_CP053100	Acinetobacter baumannii
NZ_AKBU01000003*	Cereibacter sphaeroides	NZ_CP059042	Acinetobacter baumannii
NZ_AKBU01000004	Cereibacter sphaeroides	NZ_CP074709	Acinetobacter baumannii
NZ_AKBU01000005	Cereibacter sphaeroides	NZ_CP074708	Acinetobacter baumannii
NZ_AKBU01000010	Cereibacter sphaeroides	NZ_CP074707	Acinetobacter baumannii
NZ_AKBU01000006*	Cereibacter sphaeroides	NZ_CP074712	Acinetobacter baumannii
NZ_AKBU01000011	Cereibacter sphaeroides	NZ_CP074711	Acinetobacter baumannii
NZ_AKBU01000007*	Cereibacter sphaeroides	NZ_CP068794	Deinococcus radiodurans
NZ_AKBU01000009	Cereibacter sphaeroides	NZ_CP068793*	Deinococcus radiodurans

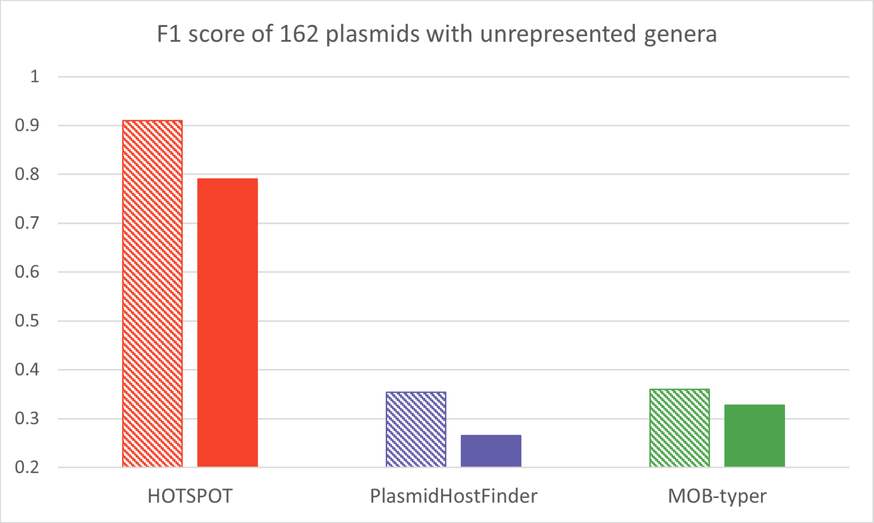

Figure 7: The prediction performance of each tool on the 162 plasmids with unknown genus-level host taxa. The patterned bars represent the F1-score at the order level, and the solid bars represent the F1-score at the family level.

8 Experiments on the novel plasmids

8.1 Experiments on the plasmids with unknown host taxa

When using HOTSPOT on the test set, there are 162 plasmids with known family-level host taxa (54 families) but unknown genus-level taxa (104 genera). Similarly, there are 734 plasmids with known genus-level taxa (137 genera) but unknown species-level taxa (437 species). Thus, we do not include these plasmids when comparing each tool's performance on the RefSeq datasets. In this section, we will consider these plasmids novel because their genus-level or species-level taxa cannot be predicted. We thus focus on reporting the prediction at higher ranks for these novel plasmids.

Figure 7 and Figure 8 show the benchmark results on the two groups of plasmids, respectively. We calculate the F1-score at the two higher taxonomic levels adjacent to the unknown level. For example, we compare each tool's performance on the order and family levels for the 162 plasmids with unknown genus-level labels. Overall, HOTSPOT performs best among the two experiments, proving that HOTSPOT can reliably predict higher-rank host taxa for the plasmids with novel taxonomic labels at lower ranks.

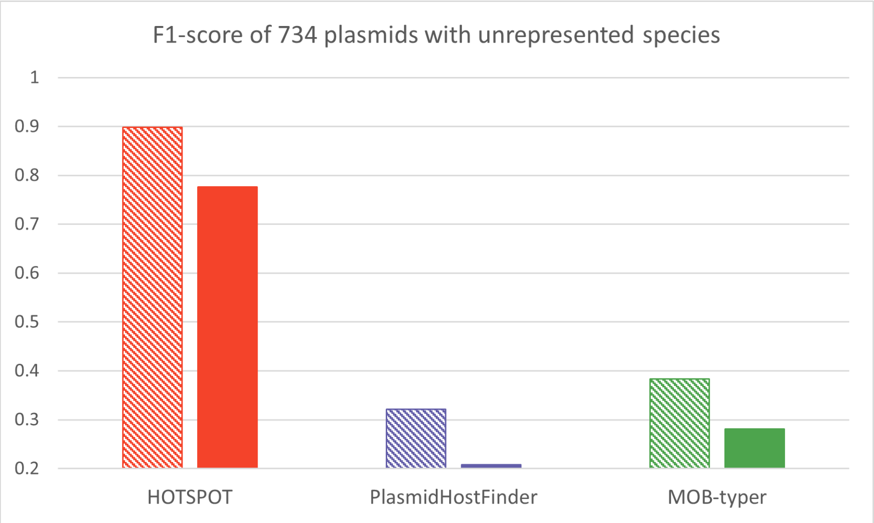

Figure 8: The prediction performance of each tool on the 734 plasmids with unknown species-level host taxa. The patterned bars represent the F1-score at the family level, and the solid bars represent the F1-score at the genus level.

8.2 Experiments on the plasmids with low similarity to the training data

In this section, we will explore how train-vs-test similarity affects plasmid host prediction. First, we use Mash [16] to implement all-against-all comparisons between complete plasmids in the training and test set. Then, we record the largest similarity against the training set for each test plasmid. From the similarity profile, we observe that all similarities smaller than 70% (corresponding to 102 test plasmids) are 0% because these plasmids do not share any 21-mer with the 21,212 training plasmids. Then, all the other plasmids have Mash similarity above 70%. Thus, we select four similarity cutoffs, 70%, 75%, 80%, and 85%, and divide all test plasmids with similarity smaller than the four cutoffs into four groups. The F1-score benchmark results of the four groups are shown in the table below. The two numbers in the brackets in the first row represent the average Mash similarity against the training set and the number of plasmids in that group. As shown in the table, HOTSPOT outperforms the other three tools on the four groups of plasmids, suggesting that HOTSPOT is more reliable on plasmids with low similarity to the training data.

Ranks	Tools	<70% (0%, 102)	<75% (47.5%, 306)	<80% (56.8%, 446)	<85% (62.9%, 584)
Phylum	HOTSPOT	0.9118	0.9444	0.9574	0.9623
	PlasFlow	0.7451	0.7908	0.8206	0.8305
	PlasmidHostFinder	0.5196	0.5588	0.6009	0.6199
	MOB-typer	0.0	0.0327	0.0673	0.1233
Class	HOTSPOT	0.7113	0.8384	0.8764	0.8974
	PlasmidHostFinder	0.4021	0.4175	0.4508	0.473
	MOB-typer	0.0	0.0303	0.0572	0.1026
Order	HOTSPOT	0.5223	0.7482	0.7981	0.8391
	PlasmidHostFinder	0.1977	0.2299	0.292	0.3126
	MOB-typer	0.0	0.0292	0.0511	0.0987
Family	HOTSPOT	0.4493	0.6125	0.6791	0.7217
	PlasmidHostFinder	0.2029	0.1958	0.2299	0.2386
	MOB-typer	0.0	0.0333	0.0481	0.0815
Genus	HOTSPOT	0.3333	0.5141	0.5663	0.6107
	PlasmidHostFinder	0.1765	0.1695	0.1935	0.1985
	MOB-typer	0.0	0.0226	0.0215	0.0458
Species	HOTSPOT	0.0714	0.2273	0.3333	0.4
	PlasmidHostFinder	0.0714	0.1818	0.2619	0.2769

9 The F1-score benchmark results on the short plasmid contigs

Taxonomic rank	Tools	1.5kbp	3kbp	5kbp	10kbp
Phylum	HOTSPOT	0.9691	0.9742	0.9783	0.985
	PlasFlow	0.6003	0.768	0.8611	0.9361
	PlasmidHostFinder	0.8789	0.8742	0.8768	0.8778
	MOB-typer	0.202	0.2809	0.3498	0.5018
Class	HOTSPOT	0.9353	0.9491	0.9555	0.9662
	PlasmidHostFinder	0.831	0.8272	0.8238	0.8215
	MOB-typer	0.1956	0.2724	0.3385	0.4886
Order	HOTSPOT	0.8894	0.9105	0.9193	0.9367
	PlasmidHostFinder	0.7317	0.7246	0.7214	0.7189
	MOB-typer	0.1699	0.2446	0.3092	0.4507
Family	HOTSPOT	0.8497	0.8732	0.8836	0.9025
	PlasmidHostFinder	0.6861	0.6767	0.6732	0.6716
	MOB-typer	0.1474	0.2104	0.2601	0.3757
Genus	HOTSPOT	0.6803	0.7114	0.7344	0.7557
	PlasmidHostFinder	0.5101	0.5023	0.5025	0.4955
	MOB-typer	0.0739	0.1199	0.1563	0.2272
Species	HOTSPOT	0.5927	0.6199	0.6447	0.6597
	PlasmidHostFinder	0.4956	0.4922	0.4942	0.492

10 Experiments on the simulated metagenomic dataset

In this experiment, we will validate HOTSPOT on predicting the host for plasmid contigs assembled from the simulated metagenomic data. Figure 9 shows the F1-score of all tools at the six taxonomic levels. After preprocessing, the dataset contains 106 well-annotated plasmid contigs whose originating plasmids are not in our training set. Half the plasmid contigs' species-level taxa are not in our label set. This indicates that many novel plasmids exist in this dataset and are

highly different from our training set. Nevertheless, HOTSPOT outperforms the other three approaches on this hard case, which suggests that HOTSPOT is more reliable with complex metagenomic data.

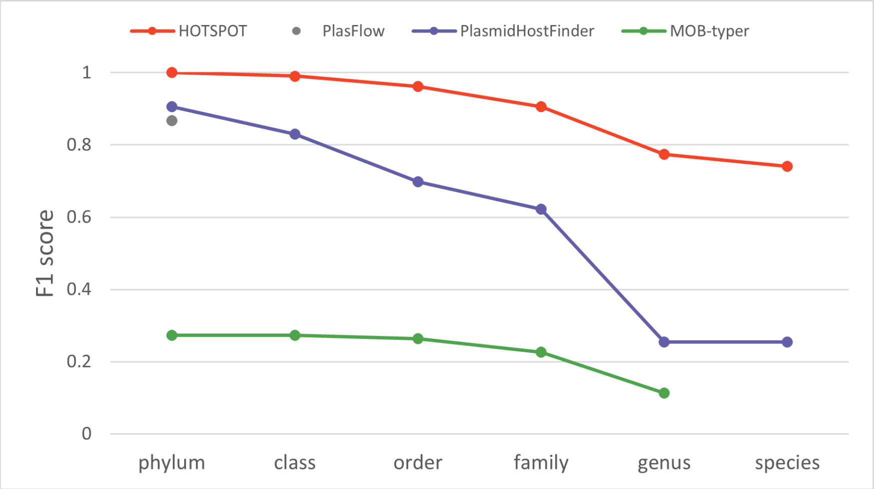

Figure 9: The prediction performance on the simulated metagenomic dataset. X-axis: the six taxonomic ranks. Y-axis: the F1-score.

11 Comparison between the results by HOTSPOT and Kraken 2

Because the Hi-C dataset is a complex real-life dataset, we can also employ another manner to test HOTSPOT's performance on plasmid host prediction. We first input Kraken 2 with the raw Hi-C reads to get the comprehensive taxonomic profiling of the potential bacterial hosts. The taxa with an estimated relative abundance smaller than 1e-5 will be removed. Then, we can compare the remaining taxa with the host taxa predicted by HOTSPOT on the 501 identified plasmid contigs. Ideally, the host taxa by HOTSPOT should be the subset of the taxa classified by Kraken 2 because not all bacteria in the sample carry plasmids. The comparison results are shown in Table 5. We can observe that HOTSPOT's results are the subset of taxa classified by Kraken 2 from phylum to genus level. Nevertheless, there are three false positives by HOTSPOT at the species level, *Neisseria gonorrhoeae*, *Paracoccus marcusii*, and *Enterobacter cloacae* complex sp., respectively. A closer look at Kraken 2's sample report shows that 1,479, 1,500, and 591 reads are assigned to the three species, respectively, but the three species are removed because of the low relative abundances. This experiment reveals that HOTSPOT can be generalized to complex real-life metagenomic data.

Ranks	HOTSPOT	Kraken 2	Shared taxa
Phylum	4	19	4
Class	7	35	7
Order	16	81	16
Family	20	179	20
Genus	26	390	26
Species	41	682	38

Table 5: The first two columns represent the number of classified taxa by HOTSPOT and Kraken 2 on the six taxonomic ranks. The third column represents the number of shared taxa by HOTSPOT and Kraken 2.

12 Experiments on the CAMI2 marine dataset

Table 6: The prediction results on the CAMI2 marine S0 dataset. (#) in column 1: the number of annotated plasmid contigs at different ranks. The numbers in other columns: the correctly predicted plasmid contigs.

Ranks (# of provided plasmid contigs)	HOTSPOT-sensitive	PlasmidHostFinder-sensitive	MOB-typer
Phylum (1127)	847	642	31
Class (1127)	762	224	28
Order (1108)	519	14	22
Family (1060)	444	2	17
Genus (749)	273	3	14
Species (252)	142	1	-

We use the contigs annotated as "plasmid" in the CAMI2 marine S0 dataset as the inputs to all tools. Table 6 shows the number of correctly predicted plasmids of the three tools for each rank. The decrease of the annotated contigs (i.e., plasmids) at the genus and species level indicates that a large portion of the contigs in the CAMI2 datasets are novel and not reported in the RefSeq database. As a result, MOB-typer and PlasmidHostFinder can only correctly predict very few contigs, especially at the genus and species level. In contrast, the accuracy of HOTSPOT is still much better than the state-of-the-art methods.

References

[1] Masaki Shintani, Zoe K Sanchez, and Kazuhide Kimbara. Genomics of microbial plasmids: classification and identification based on replication and transfer systems and host taxonomy. *Frontiers in microbiology*, 6:242, 2015.

[2] M Pilar Garcillán-Barcia, Santiago Redondo-Salvo, Luis Vielva, and Fernando de la Cruz. Mobscan: automated annotation of mob relaxases. In *Horizontal Gene Transfer*, pages 295–308. Springer, 2020.

[3] Alessandra Carattoli, Ea Zankari, Aurora García-Fernández, Mette Voldby Larsen, Ole Lund, Laura Villa, Frank Møller Aarestrup, and Henrik Hasman. In silico detection and typing of plasmids using plasmidfinder and plasmid multilocus sequence typing. *Antimicrobial agents and chemotherapy*, 58(7):3895–3903, 2014.

[4] Nicholas J Dimonaco, Wayne Aubrey, Kim Kenobi, Amanda Clare, and Christopher J Creevey. No one tool to rule them all: prokaryotic gene prediction tool annotations are highly dependent on the organism of study. *Bioinformatics*, 38(5):1198–1207, 2022.

[5] Benjamin Buchfink, Chao Xie, and Daniel H Huson. Fast and sensitive protein alignment using diamond. *Nature methods*, 12(1):59–60, 2015.

[6] William R Pearson. An introduction to sequence similarity ("homology") searching. *Current protocols in bioinformatics*, 42(1):3–1, 2013.

[7] Kaiming He, Xiangyu Zhang, Shaoqing Ren, and Jian Sun. Deep residual learning for image recognition. In *Proceedings of the IEEE conference on computer vision and pattern recognition*, pages 770–778, 2016.

[8] Jimmy Lei Ba, Jamie Ryan Kiros, and Geoffrey E Hinton. Layer normalization. *arXiv preprint arXiv:1607.06450*, 2016.

[9] Daniel N Baker and Ben Langmead. Dashing: fast and accurate genomic distances with hyperloglog. *Genome biology*, 20(1):1–12, 2019.

[10] Lucas R van Dijk, Bruce J Walker, Timothy J Straub, Colin J Worby, Alexandra Grote, Henry L Schreiber IV, Christine Anyansi, Amy J Pickering, Scott J Hultgren, Abigail L Manson, et al. Strainge: a toolkit to track and characterize low-abundance strains in complex microbial communities. *Genome biology*, 23(1):74, 2022.

[11] James Robertson and John HE Nash. Mob-suite: software tools for clustering, reconstruction and typing of plasmids from draft assemblies. *Microbial genomics*, 4(8), 2018.

[12] Oliver Schwengers, Patrick Barth, Linda Falgenhauer, Torsten Hain, Trinad Chakraborty, and Alexander Goesmann. Platon: identification and characterization of bacterial plasmid contigs in short-read draft assemblies exploiting protein sequence-based replicon distribution scores. *Microbial genomics*, 6(10), 2020.

[13] Nils Hülter, Judith Ilhan, Tanita Wein, A Samer Kadibalban, Katrin Hammerschmidt, and Tal Dagan. An evolutionary perspective on plasmid lifestyle modes. *Current opinion in microbiology*, 38:74–80, 2017.

[14] Finlay Maguire, Baofeng Jia, Kristen L Gray, Wing Yin Venus Lau, Robert G Beiko, and Fiona SL Brinkman. Metagenome-assembled genome binning methods with short reads disproportionately fail for plasmids and genomic islands. *Microbial genomics*, 6(10), 2020.

[15] Qichao Tu, Zhili He, and Jizhong Zhou. Strain/species identification in metagenomes using genome-specific markers. *Nucleic acids research*, 42(8):e67–e67, 2014.

[16] Brian D Ondov, Todd J Treangen, Páll Melsted, Adam B Mallonee, Nicholas H Bergman, Sergey Koren, and Adam M Phillippy. Mash: fast genome and metagenome distance estimation using minhash. *Genome biology*, 17(1):1–14, 2016.
